# Short and narrow flag leaf1, a GATA zinc finger domain-containing protein, regulates flag leaf size in rice (*Oryza sativa*)

**DOI:** 10.1186/s12870-018-1452-9

**Published:** 2018-11-09

**Authors:** Peilong He, Xiaowen Wang, Xiaobo Zhang, Yudong Jiang, Weijiang Tian, Xiaoqiong Zhang, Yangyang Li, Ying Sun, Jia Xie, Jile Ni, Guanghua He, Xianchun Sang

**Affiliations:** 1 Key Laboratory of Application and Safety Control of Genetically Modified Crops, Rice Research Institute of Southwest University, Academy of Agricultural Sciences, Southwest University, Chongqing, China; 2 Key Laboratory of Southwest Rice Biology and Genetic Breeding, Institute of Rice and Sorghum, Sichuan Academy of Agricultural Sciences, Deyang, China

**Keywords:** Rice (*Oryza sativa*), Alternative splicing, Short and narrow leaf, Map-based cloning, GATA zinc finger domain

## Abstract

**Background:**

The flag leaf of rice (*Oryza sativa* L.) is an important determinant of plant type characteristics and grain yield. Identification of flag leaf mutants of rice is crucial to elucidate the molecular mechanism of flag-leaf development, and for exploitation of rice germplasm resources.

**Results:**

In this study, we describe a mutant designated *short and narrow flag leaf 1* (*snfl1*). Histological analysis showed that the length of epidermal cells and number of longitudinal veins were decreased in the flag leaf of the *snfl1* mutant. Map-based cloning indicated that a member of the GATA family of transcription factors is a candidate gene for *SNFL1*. A single-nucleotide transition at the last base in the single intron of *snfl1* led to variation in alternative splicing and early termination of translation. Complemented transgenic plants harbouring the candidate *SNFL1* gene rescued the *snfl1* mutant. Analysis of RT-PCR and the *SNFL1* promoter by means of a GUS fusion expression assay showed that abundance of *SNFL1* transcripts was higher in the culm, leaf sheath, and root. Expression of the SNFL1-GFP fusion protein in rice protoplasts showed that SNFL1 was localized in nucleus.

**Conclusions:**

We conclude that *SNFL1* is an important regulator of leaf development, the identification of which might have important implications for future research on GATA transcription factors.

**Electronic supplementary material:**

The online version of this article (10.1186/s12870-018-1452-9) contains supplementary material, which is available to authorized users.

## Background

Rice is one of the most important cereal crops in the world and is also the staple food for nearly 50% of the world’s population [1]. However, the total area of arable land is decreasing concurrent with rapid industrial development, therefore maintenance or increase in the yield of rice is strongly dependent on the improvement of grain yield per acre. Improving plant type is essential in increasing this yield. As a model monocotyledonous plant, the elucidation of physiological and biochemical mechanisms in rice are important for research on cereals and monocotyledonous species.

Among the rice and other major cereals, the uppermost three leaves, especially the flag leaf, are the main source of the carbohydrates that eventually accumulate in the grains [2–5]. Previous studies of rice leaf phenotypes have focused on mapping of quantitative trait loci, and relatively few genes responsible for leaf phenotypes have ever been cloned. Rice leaf elongation is mostly determined by longitudinal cell division, cell elongation, and cell arrangement. For example, leaves of the rice dwarf mutant *dwarf and gladius leaf 1* (*dgl1*) are shorter and the edges of the leaf tips more rounded than those of the wild type (WT). The reason of the abnormal phenotype of *dgl1* is that the longitudinal epidermal cells are not well elongated and the cells are distorted and bulky, leading to an abnormal cell file. [6]. An additional rice mutant with an aberrant leaf length, *Oryza sativa ent-kaurene synthase 2* (*osks2*), produces short and dark green leaves in the seedling stage. The short leaves of *osks2* is a result of the abnormal arrangement of the longitudinal mesophyll cells of osks2 leaves, which were packed more tightly than in the WT [7]. Overexpression of *Oryza sativa PATATIN-RELATED PHOSPHOLIPASE A IIIα* (*OspPLAIIIα*) results in the decreased length of rice leaves. *OspPLAIIIα* plays an important role in rice vegetative growth. Furthermore, high activity of OspPLAIII can suppress cell elongation [8]. The rice mutant *brassinosteroid-dependent 1* (*brd1*) produces short leaves, the mesophyll cells of *brd1* leaf blades are arranged more tightly than those of the WT, and intercellular spaces in the leaf blade of *brd1* plants are smaller than those of the WT. In addition, motor cells and epidermal cells along the longitudinal axis of the *brd1* leaf blades are much shorter than WT cells. All of these traits contribute to the short-leaf phenotype [9].

Variations in rice leaf width are mainly due to changes in vascular bundles and cell division. Many genes responsible for narrow leaf phenotypes have been cloned, such as *NARROW LEAF 1* (*NAL1*) [10], *NAL2* [11], *NAL3* [11], *NAL7* [12], *NARROW AND ROLLED LEAF 1* (*NRL1*) [13], and *ABNORMAL VASCULAR BUNDLES* (*AVB*) [14]. Leaves of the *nal1* mutant are narrower than those of the WT, and the cause of the abnormal phenotype was indicated to be a decrease in the number of longitudinal veins. *NAL1* affects vascular patterns of rice and polar auxin transport, and plays an important role in controling lateral leaf growth [10]. The *nal2/3* double mutant produces narrow-curly leaves, which result mainly from fewer longitudinal veins and reduced lateral-axis outgrowth. *NAL2* and *NAL3* are paralogs that encode an identical WUSCHEL-RELATED HOMEOBOX 3A (OsWOX3A; OsNS) transcriptional activator, which is involved in lateral-axis outgrowth and vascular patterning in leaves, organ development, development of tillers and lateral roots in rice, and lemma and palea morphogenesis in spikelets [11]. The *nrl1* mutant exhibits a reduced leaf-width phenotype. Microscopic analysis indicates that leaves of the *nrl1* mutant have fewer longitudinal veins compared with the WT [13]. The *avb* mutant causes narrow leaves. The reason of the abnormal phenotype of *avb* leaves is that the number of vascular bundles in the aerial organs is reduced. *AVB* plays an important role in maintenance of the normal cell division in lateral primordia development. [14].

In the present study, we identified a *short and narrow flag leaf 1* (*snfl1*) mutant of rice and isolated the gene responsible using a map-based cloning strategy. *SNFL1* encodes a GATA zinc finger domain-containing protein. The present findings indicate the involvement of *SNFL1* in the development of the epidermal cells, longitudinal veins, panicle length, number of panicles, and 1000-grain weight, demonstrating the numerous important roles of this gene in rice growth and development.

## Results

### Phenotypes of the *snfl1* mutant

**Fig. 1:**
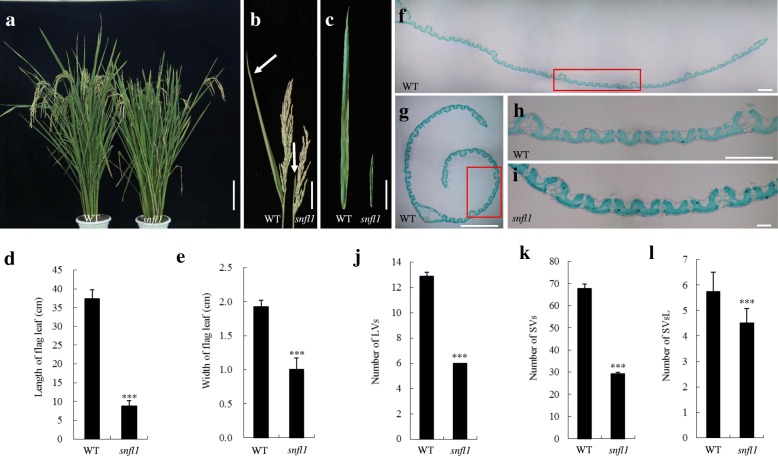
Phenotype of the *snfl1* mutant. (**a**) Gross morphology of the wild type (WT) and *snfl1* mutant at the grain filling stage. (Scale bar, 20 cm.) (**b**) Panicle and flag leaf of the WT and *snfl1* at the grain filling stage. Arrows indicate the flag leaf. (Scale bar, 5 cm.) (**c**) Flag leaf of WT and *snfl1*. (Scale bar, 5 cm.) (**d**,**e**) Flag leaf length (**d**) and flag leaf width (**e**) in the WT and *snfl1*. (**f**,**g**) Transverse sections of the flag leaf of the WT (**f**) and *snfl1* (**g**). (Scale bars, 1 mm.) (**h**,**i**) Transverse sections of the flag leaf of the WT (**h**) and *snfl1* (**i**) between two large vascular bundles. (Scale bars, 400 μm.) (**j**–**l**) Number of large vascular bundles (LVs) (**j**), number of small vascular bundles (SVs) (**k**), and number of small vascular bundles between two large vascular bundles (SVsL) (**l**) in the WT and *snfl1*. Data are the mean ± SD (*n* = 10). ***P < 0.001 (Student’s *t*-test)

To investigate the molecular mechanisms that regulate rice leaf development, we used ethyl methanesulfonate (EMS) mutagenesis to generate a population of M_2_ seedlings derived from 1 kg of rice seeds (about 40 thousand) from the cultivar ‘Jinhui10’. We screened one leaf of mutants showing an altered leaf size, which had been continuously planted for many generations, and for which the mutant traits were stable and inherited. One such mutant, *snfl1*, was selected for detailed study. The absence of a significant difference in plant height between WT and *snfl1* (Fig. 1a; Additional file 1: Figure S1). However, the flag leaf length of *snfl1* was dramatically decreased by about 83.28%, and the flag leaf width decreased by about 67.69% compared with the WT (Fig. 1b–e). Similarly, the length of the second and third leaves of *snfl1* were decreased, whereas the width of the second leaf showed no significant difference, and the width of the third leaf was markedly increased, compared with those of the WT (Additional file 1: Figure S1). In addition, the *snfl1* mutant exhibited other abnormal phenotypes, such as reduced panicle length, decreased numbers of primary and secondary panicles, reduced number of grains per main panicle, seed setting rate and 1000-grain weight, and overgrown culms (Fig. 1b; Table 1 and Additional file 1: Figure S1). Thus, the *snfl1* mutant showed multiple morphological defects.

**Table 1:** Agronomic traits of the wild type (WT) and the *snfl1* mutant

Traits	WT	*snfl1*
Plant height (cm)	117.63 ± 1.74	118.16 ± 1.23
Effective panicle number per plant	10.00 ± 1.20	9.33 ± 1.53
Panicle length (cm)	27.10 ± 0.91	22.76 ± 1.30^***^
Number of primary panicle	11.38 ± 1.30	8.80 ± 0.84^**^
Number of second panicle	38.50 ± 3.07	28.60 ± 3.44^***^
Filled grain number per panicle	150.00 ± 16.44	95 ± 15.38^***^
Seed setting rate (%)	89.01 ± 1.35	63.79 ± 0.04^***^
1000-grain weight (g)	26.25 ± 0.48	24.38 ± 0.68^**^

** means significantly different compared with the corresponding WT at *p* < 0.01 and *** means significantly different compared with the corresponding WT at *p* < 0.001 by the Student’s *t* test

### Histological analysis of *snfl1* flag leaf

To investigate the basis for the narrow flag leaf of the *snfl1* mutant, we examined the flag leaf anatomy. Compared with the WT, the number of large vascular bundles per flag leaf was reduced by almost half and the number of small vascular bundles per flag leaf was reduced by 43.3% in the *snfl1* mutant. In addition, the number of small vascular bundles between adjacent large vascular bundles was reduced to 21.3% of that in the WT (Fig. 1f-l). These results indicate that the narrow flag leaf phenotype should result from the reduced number of vascular bundles in *snfl1*.

**Fig. 2:**
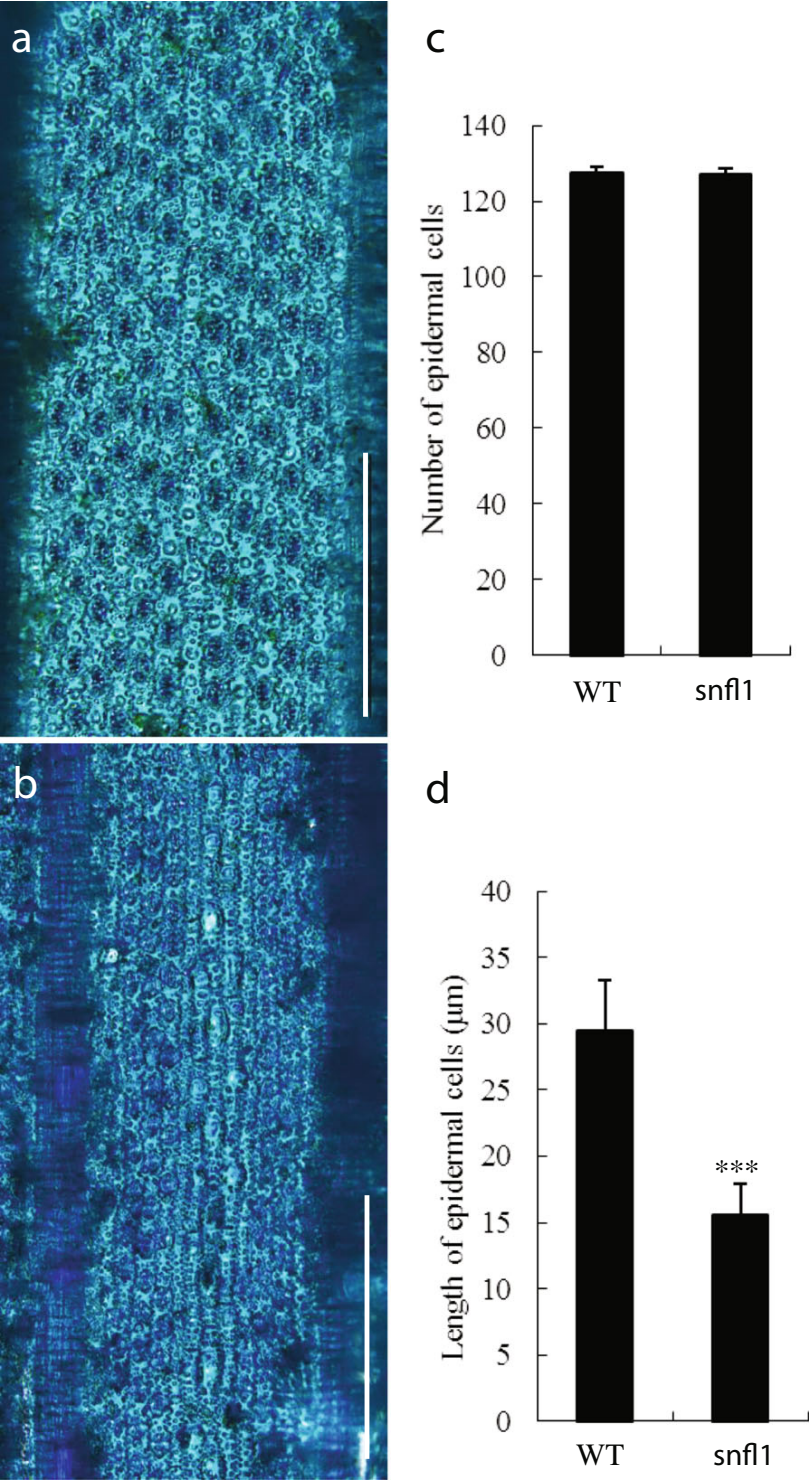
Histological analysis of the flag leaf. (**a**) Adaxial epidermal peels of WT mature flag leaves. (Scale bar, 200 μm.) (**b**) Adaxial epidermal peels of *snfl1* mature flag leaves. (Scale bar, 200 μm.) (**c**,**d**) Number of epidermal cells (**c**) and length of epidermal cells (**d**) in the WT and *snfl1*. Means ± SD are given in C (*n* = 10), and D (*n* = 60). ***P < 0.001 (Student’s *t*-test)

To examine the detailed anatomy of the short leaves in *snfl1*, the epidermis of the leaf was peeled off after cellulase digestion and stained by Toluidine Blue O. The results showed that the number of epidermal cells in the adaxial epidermis of the flag leaf had no obvious difference between the WT and *snfl1* mutant. However, the lengths of the epidermal cells of *snfl1* were dramatically decreased compared with those of the WT (Fig. 2a–d).

These observations indicate that the decreased length of the epidermal cells and the decreased number of vascular bundles might be associated with the short, narrow flag leaf in the *snfl1* mutant.

### Map-based cloning of *SNFL1*

**Fig. 3:**
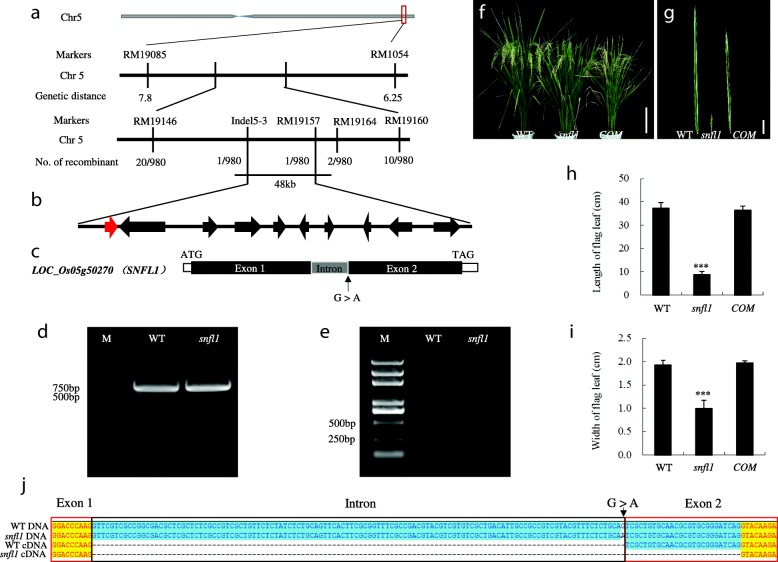
Map-based cloning of *snfl1*. (**a**) Positional cloning of *snfl1*. The *snfl1* gene was mapped to a 48-kb genomic region between the markers Ind5–3 and RM19157. The marker names are above the vertical lines; the genetic distance and the number of recombinants are displayed below the vertical lines. (**b**) Ten candidate genes localized between Ind5–3 and RM19157. (**c**) Gene structure and mutation site of the candidate gene *LOC_Os05g50270* (*SNFL1*). Black boxes indicate exons and grey boxes represent the intron. *snfl1* contains one point mutation (G to A) in the last base of the single intron. (**d**,**e**) Verification of the mutation site by enzyme digestion. (**d**) PCR verification of the mutation site before enzyme digestion. (**e**) PCR verification of the mutation site after enzyme digestion. (**f**–**i**) Complementation test of *snfl1*. (**f**) Gross morphology of the WT, *snfl1* mutant, and complemented transgenic plant (*COM*) at the grain filling stage. (Scale bar, 20 cm.) (**g**) Flag leaf of the WT, *snfl1* mutant, and *COM* at the grain filling stage. (Scale bar, 5 cm.) (**h**,**i**) Flag leaf length (**h**) and flag leaf width (**i**) in the WT, *snfl1* mutant, and *COM* at the grain filling stage. (**j**) Alignment of DNA and cDNA between the WT and *snfl1*. Red boxes indicate exons, black box indicates the intron. Black arrows represent the mutation site. The alignment was generated using Vector NTI 10 software

We isolated the *SNFL1* gene using a positional cloning method. A genetic population was established from a cross between the *snfl1* mutant and *japonica* rice Nipponbare. All F_1_ hybrids derived from the cross showed a normal phenotype similar to that of Nipponbare. In the F_2_ population, of 4192 F_2_ individual plants investigated, 980 exhibited the *snfl1* mutant phenotype and 3212 exhibited the WT phenotype. The segregation ratio conformed to a 3:1 ratio (${\upchi}_{0.05}^2$= 2.72 < ${\upchi}_{0.05}^2$= 3.84), which indicates that the *snfl1* phenotype is controlled by a single recessive nuclear gene. To map the *SNFL1* locus, plants showing the *snfl1* mutant phenotype were sampled to carry out linkage analysis in the F_2_ population using the bulked segregant analysis method [15]. Rough mapping localized the *SNFL1* locus to a 14.25-cM region (about 1.6 Mb) on the long arm of chromosome 5 delimited by the simple sequence repeat (SSR) markers RM19085 and RM1054 (Fig. 3a). For fine mapping of *SNFL1*, a suite of Indel and SSR markers were newly designed based on genomic sequence differences between Nipponbare and Jinhui10. Genotyping of 980 F_2_ mutant individuals revealed that *SNFL1* was localized to a 48-kb region between the Indel marker Indel5–3 and the SSR marker RM19157 (Fig. 3a).

According to the Gramene database (http://www.gramene.org), the 48-kb region contains ten putative open reading frames (ORFs) (Fig. 3b). Among the 10 ORFs sequenced, the *snfl1* mutant contained a single-nucleotide transition (G → A), in the only intron at the last base sequence, at the locus *LOC_Os05g50270* (Fig. 3c and j). We thus hypothesize that the putative gene *LOC_Os05g50270* was the candidate gene of *SNFL1*. Given that a *Pst*I restriction enzyme cutting site was present at the mutation site (Additional file 2: Figure S2), we carried out enzyme digestion at the mutation site for the mutant and WT plants. The enzyme digestion products were detected by 1% agarose gel electrophoresis. This analysis demonstrates that the second and third fragment in the mutants could not be digested and resulted in a longer fragment than that of the WT (Fig. 3d-e). This result provides further evidence that the locus *LOC_Os05g50270* represents the target gene *SNFL1*.

The mutation may lead to abnormal splicing of the intron in the *snfl1* mutant. To verify this hypothesis, we sequenced the cDNA of *SNFL1* of the WT and *snfl1* mutant. Sequence verification of the positive clones identified from the cDNA libraries demonstrated that the WT cDNA contained an 840-bp exon generated by proper splicing of the intron. In contrast, the *snfl1* cDNA contained an 814-bp exon resulting from deletion of 26 bp in second exon (Fig. 3j). The deletion led to early termination of translation. Therefore, the altered intron in *snfl1*, with the 26-bp deletion in the second exon, is considered to be responsible for the short and narrow flag leaf phenotype in the *snfl1* mutant.

To confirm that *SNFL1* disruption resulted in the abnormal leaf phenotype, we performed a complementation experiment. The positive plants were detected using a β-glucuronidase (GUS) array analysis. As expected, the short, narrow leaf phenotype was not observed among the positive plants, all of which showed the normal leaf phenotype. Sequence verification of *SNFL1* in the complemented transgenic plants revealed the presence of two peaks at the mutation site (Fig. 3f-i; Additional file 3: Figure S3). These results confirmed that *LOC_Os05g50270* corresponds to *SNFL1* and the *snfl1* mutant harbours defective *SNFL1*.

### Phylogenetic analysis of SNFL1 homologs

**Fig. 4:**
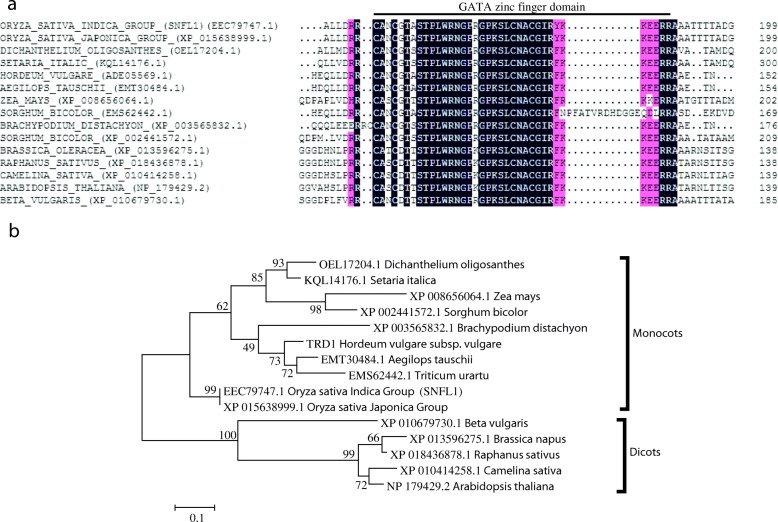
Multiple-sequence alignment and phylogenetic relationships of SNFL1 with homologous protein sequences. (**a**) Multiple-sequence alignment of sequences for SNFL1 and other GATA domain-containing proteins. The alignment of GATA domain-containing proteins was generated with ClustalX2.1 software. Dark blue shading indicates 100% similarity of the aligned sequence; pink shading indicates positions conserved in > 75% of the aligned sequences. (**b**) Phylogenetic tree for SNFL1 and GATA domain-containing homologs of high similarity constructed using MEGA 5.05 software

To further clarify the structure and possible function of *SNFL1*, a bioinformatics analysis was carried out. Multiple-sequence alignment and phylogenetic analysis indicated that SNFL1 consists of 280 amino acids and contains a GATA zinc finger domain. A BLAST search (https://blast.ncbi.nlm.nih.gov/Blast.cgi) revealed that SNFL1 showed high similarity with the conserved GATA zinc finger domain of *Hordeum vulgare*, *Zea mays*, *Beta vulgaris*, and *Brassica napus* (Fig. 4a and b). Among the proteins included in the analysis, only TRD1 of *Hordeum vulgare* has been studied previously. The *trd1* mutant fails to suppress bract growth and as a result produces leaf-like structures that subtend each rachis node in the basal portion of the spike, which represents the third outer glume [16]. This phenotype is similar to the *snfl1* mutant, which displayed excessive vegetative growth with overgrown culms from internodes. However, *TRD1* has not been cloned, so the underlying molecular mechanism remains unclear.

### Analysis of tissue-specific expression of *SNFL1*

**Fig. 5:**
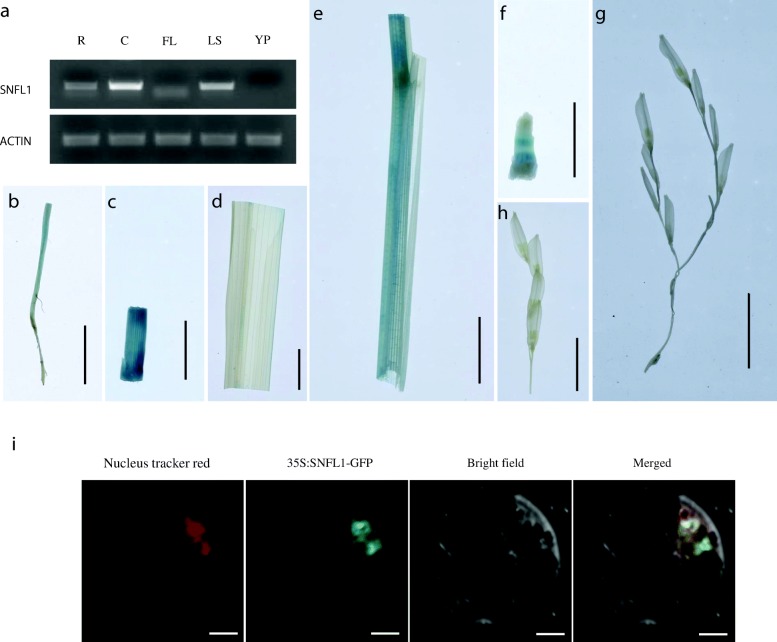
*SNFL1* expression pattern and protein subcellular localization. (**a**) RT-PCR analysis of *SNFL1* transcripts in different rice tissues. Roots (R) and culms (**c**) were harvested from 2-month-old WT plants. Flag leaf (FL), leaf sheaths (LS) and young panicles (YP) were collected at the booting stage. (**b**–**h**) GUS staining of PRO_*SNFL1*_:GUS transgenic line tissues. (Scale bar, 1 cm.) (**b**) Root. (**c**) Culm. (**d**) Leaf blade. (**e**) Leaf sheath. (**f**–**h**) Young panicles of different lengths. (**i**) Subcellular localization of SNFL1-GFP fusion protein in rice protoplasts. (Scale bar, 5 μm)

To examine the expression pattern of *SNFL1*, we detected *SNFL1* transcripts in different WT tissues at different developmental stages using RT-PCR. The abundance of *SNFL1* transcripts was highest in the culm. Furthermore, higher quantities of *SNFL1* transcripts were detected in the leaf sheath and root than in other tissues (Fig. 5a). Consistent with this result, promoter-reporter constructs revealed that *SNFL1* was predominantly expressed in the culm, leaf sheath and root of transgenic plants (Fig. 5b–h).

### Subcellular localization of SNFL1

To determine the subcellular localization of SNFL1, we fused the green fluorescent protein (GFP) gene to the ORF of SNFL1 driven by the CaMV 35S promoter and transformed the construct into rice protoplasts. The GFP signal was visualized within the protoplast cells, and colocatized with the nucleus-specific stain Nucleus Tracker Red. The co-localization supports the prediction that SNFL1 is a nuclear protein (Fig. 5i).

## Discussion

### The short and narrow flag leaves phenotype of *snfl1* should result from the abnormal development of epidermal cells and longitudinal veins

Flag leaf length has long been recognized as an important factor that determines plant type for high-yield potential in rice [17]. Elucidation of the genes associated with flag leaf development is essential for clarifying the mechanism that regulates leaf length. However, until recently few genes associated with flag leaf length had been cloned. Analysis of a large number of studies suggests that leaf development is a complex process containing cell expansion and division, axis determination, and tissue differentiation and specification [18]. Thus, cell division and expansion of mesophyll cells, especially the longitudinal cells, may affect leaf length. In the present study, histological analysis revealed that the length of epidermal cells in the flag leaf of the *snfl1* mutant was dramatically decreased compared with that of the WT (Fig. 2a–d). Therefore, the short flag leaf phenotype of *snfl1* may result from abnormal development of the epidermal cells.

Narrow flag leaves result mainly from reduced lateral-axis outgrowth and fewer longitudinal veins in rice [11, 19]. A number of genes associated with the development of longitudinal veins in rice have been studied. For example, the *nal1* mutant exhibits a significant phenotype of narrow leaves. In accordance with the narrow leaves, *nal1* leaves contain a decreased number of longitudinal veins. *NAL1* affects vascular pattern of rice and polar auxin transport, and plays an important role in lateral leaf growth [10]. In addition, the narrow leaf phenotypes of the *nal2*, *nal3*, *nrl1*, *nrl2*, and *avb* mutants may be due to reduced lateral-axis outgrowth with fewer longitudinal veins [11, 13, 20]. In the present study, the *snfl1* mutant phenotype also involved changes to the longitudinal veins. The number of longitudinal veins was distinctly decreased in the flag leaf of the *snfl1* mutant compared with that of the WT (Fig. 1c and e). The alteration in number of longitudinal veins in the flag leaf may indicate that *SNFL1* is associated with the development of longitudinal veins. However, the *snfl1* mutant also showed reduced panicle length, number of panicles, and 1000-grain weight, which indicates that *SNFL1* has a complicated function in rice plant development.

### Alternative splicing of *SNFL1* might result in short and narrow flag leaves in *snfl1*

In eukaryotes, most encoding genes are split genes that contain introns. At least 74% multi-exon genes of human are alternatively spliced and may comprise 80–85% of such genes [21, 22]. During the process of gene expression in animals and plants, introns of split genes are excised under precise regulation, and subsequently the exons are spliced to form the mature mRNA, which is translated into a functional protein. The first splicing factor identified in plants was AtRSP31, which contains an N-terminal RNA recognition motif and a C-terminal RS domain highly enriched in arginines. As a true plant splicing factor, AtRSP31 plays an important role in splicing, similar to that of other RS splicing factors. RS domain splicing factors play crucial roles in alternative and constitutive splicing in plants [23]. An additional typical splicing factor gene is *Waxy* in rice. *Wx*^*a*^ and *Wx*^*b*^ are two functional alleles of the rice *waxy* (*wx*) locus, they are defined by the huge difference in the amount of the gene product, called Wx protein, that accumulates in mature grains. The *Wx*^*a*^ allele has a normal sequence of GT at the 5′ splice junction of the first intron and encodes a high proportion of the *Wx* transcripts in the endosperm, whereas the *Wx*^*b*^ allele has TT at the 5′ splice junction and shows a low expression level among the mature transcripts. The low expression level of *Wx*^*b*^ is a result of a single-base mutation at the 5′ splice site of the first intron. Northern blot analysis also indicates that the larger transcript consisting of the unspliced first intron is closely related to the function of the *Wx*^*b*^ allele. The *Wx*^*b*^ allele of rice carrying the G-to-T mutation of the first intron has been conserved in cultivated rice because the mutation causes low amylose content in the grain [24, 25]. Thus, mutation at splice sites of introns always leads to alternative splicing (AS), which is a crucial mechanism of post-transcriptional regulation, and can increase mRNA stability and protein diversity. Furthermore, AS plays a fundamental role in plant development, growth, and responses to external cues. The instances of AS identified in flowering plants far exceeds previous predictions, and thus its function in plants may also surpass expectations [26].

The identification of the mRNA splicing site is mainly accomplished via splicing complexes, of which there are two main kinds in higher eukaryotes. The first is the dominant U2 type splicing complex, which contains several small intranuclear complexes of U1, U2, U4, U5, and U6. The function of the U2 type splicing complex is to cut the intron of the GT-AG type splicing site. The second is the U12 type splicing complex, including several small intranuclear complexes of U11, U12, U4A, U5, and U6. The function of the U12 type splicing complex is to cut a small part of the introns [27, 28]. The introns in plants are rich in U and UA, and the exons are rich in G. This bias is crucial for the identification of the splicing sites and the efficiency of the splicing. The specific splicing mechanism remains unclear, but it is now certain that four splicing recognition signals play an important role in the normal splicing of the pre-mRNA introns. These signals are: (1) The conserved recognition sequence at the 5′ terminal of the intron is GU. (2) The conserved recognition sequence at the 3′ terminal of the intron is AG, specifically recognizable by the small nuclear U2 subunit. (3) The polypyrimidine recognizable by the small nuclear protein U2 factor AF65 at the 3′ terminal of the intron. (4) The key branch locus, the sequence features of which are conserved CURAY (R is purine, Y is pyrimidine), its position being about 17~ 40 nucleotides away from the 3′ terminal splicing site, which can be seen from the small nucleoprotein U2 Subunit [29, 30]. In the present study, we found that the intron of *SNFL1* conforms to most of the splicing rules, starting with GU and ending with AG, and the 3′ terminal can be recognized by the U2 subunit. It is worth noting that the 3′ terminal sequence of the exon fragment that is cut off is also AG, therefore the reason for the AS of *snfl1* may be the 3′ terminal mutation of the intron, making it unrecognizable by the U2 subunit. The next -AG sequence is recognizable in the 3′ terminal sequence of the exon that was cut off (Fig. 3j). The short and narrow flag leaf phenotype of *snfl1* may result from AS of the intron, and the *snfl1* mutant may thus provide valuable material for future research on alternative splicing.

### Role of GATA transcription factors in plant development

Transcription factors serve multiple functions. They can be combined with the target gene promoter and can affect positive or negative regulation of transcriptional activity. Many families of transcription factors are known in plants, such as the bZIP, bHLH, FAR1, CAMTA, GRAS, NAC, and GATA families. The GATA family has the ability to recognize GATA motifs, and most contain zinc finger structures. The GATA family is characterized by an extremely high affinity for the consensus sequence (T/A)GATA(A/G) and have been identified among fungi, metazoans, and plants [31]. GATA is a type of transcription factor that is prevalent in eukaryotes and plays an important role in biological processes, such as regulation of plant light response, chlorophyll synthesis, cytokinin response, and metabolism of carbon and nitrogen. The (T/A)GATA(A/G) sequence was first detected in the chicken globin gene promoter [32]. Subsequently, the transcription factor GATA-1 was identified in 1991, followed by the isolation of other GATA transcription factors [33–36]. GATA transcription factors are members of the zinc finger protein family, and can identify and specifically bind DNA sequences. The DNA binding domain of GATA factors constitutes a type IV zinc finger in the form of CX_2_CX_17–20_CX_2_C, followed by a highly basic region [31]. This is consistent with the present finding that SNFL1 contains a GATA zinc finger domain of the form CX_2_CX_18_CX_2_C (Fig. 4a).

GATA transcription factors are common in plants and play an important role in regulation of flowering time, leaf growth, flower development, photoperiodism and optical signal transduction. These biological processes are associated with growth and development of plants. For example, the *CO* gene was suggested to encode a protein with two zinc fingers related to those of GATA transcription factors. *CO* is associated with circadian rhythm regulation and regulation of meristems, and therefore plays an important role in regulation of flowering time by adjusting the photoperiod [37]. The *snfl1* mutant produces short and narrow leaves, which indicates that *SNFL1* may play an important role in rice leaf development. Some GATA transcription factors have been identified previously in rice. One example is *NECK LEAF1* (*NL1*), the allelic gene of *SNFL1*, in which the *nl1* mutant shows abnormal patterns of upper internode elongation, smaller panicles with overgrown bracts, and a delayed flowering time. Overexpression of *NL1* in transgenic plants usually results in severe growth retardation, smaller leaves, and fewer vegetative phytomers, which indicates that *NL1* plays an important role in organ differentiation [38]. In the present study, *snfl1* has overgrown culms and abnormal panicles (Fig. 1b and Additional file 1: Figure S1), which is similar to *nl1* and may indicate that the functions of *NL1* and *SNFL1* have much in common. On the whole, *SNFL1* encodes a GATA zinc finger protein and may have multiple functions in plant development.

## Conclusions

In this study, a gene that affects leaf length and width, which we term *short and narrow flag leaf 1* (*SNFL1*), was identified in rice using a map-based cloning strategy. The gene is located in a 48-kb genomic region between Indel5–3 and RM19157 on chromosome 5. Sequencing analysis revealed the presence of a single-nucleotide mutation (G → A) at the locus *LOC_Os05g50270*. *LOC_Os05g50270* was confirmed to correspond with *SNFL1* by transgenic complementation. *SNFL1* encodes a GATA zinc finger domain-containing protein and its loss of function led to development of short and narrow leaves. The short and narrow leaf phenotype was associated with abnormal development of the epidermal cells and longitudinal veins. *SNFL1* might have functions in multiple biological processes, such as the development of epidermal cells, longitudinal veins, and panicle development.

## Methods

### Plant material

The *snfl1* mutant with short flag leaf was isolated from the M_2_ generation of *indica* rice (*Oryza sativa L. subsp*. *indica*) ‘Jinhui 10’ by EMS treatment. The F_2_ mapping population was raised from a cross between the *snfl1* mutant and *japonica* rice (*O. sativa* subsp. *japonica*) ‘Nipponbare’. All plants were grown under natural conditions in an experimental field at the Southwest University Rice Research Institute in Chongqing, China.

### Histological analysis

The middle portion of flag leaf of WT and *snfl1* plants at the heading stage was fixed in FAA solution (70% ethanol, 5% formaldehyde and 5% acetic acid [*v*/v/v]) for 48 h. The samples were infiltrated and embedded in paraffin after dehydration in an ethanol series. The embedded samples were cut into sections 8 μm thick with a rotary microtome (RM2245, Leica Microsystems, Hamburg, Germany), stained with fast green and counterstained with safranin, and observed under a light microscope (Eclipse Ci-L, Nikon, Tokyo, Japan).

For anatomical observation of the leaf epidermis, about 1 cm from the middle portion of rice leaves was excised, placed under water, and evacuated with a vacuum pump for 30 min. The evacuated samples were digested for 48 h in 2% cellulase R-10, and stained overnight in 0.5% toluidine blue O. After washing with water, an epidermal peel was prepared and sealed under a coverslip for observation with a light microscope (Eclipse Ci-L, Nikon, Tokyo, Japan) and digital images were captured following the method of Li et al. [39].

### Measurement of agronomic traits

Five WT and five *snfl1* mutant plants were used to measure agronomic traits at the mature stage. The measured agronomic traits comprised the length and width of three functional leaves, plant height, effective panicle number per plant, panicle length, number of primary panicles, number of secondary panicles, number of filled grains per panicle, seed setting rate, and 1000-grain weight. Values presented are the means ± SD of three biological repeats. The student’s *t*-test was applied for statistical analysis of the data.

### DNA extraction and PCR analysis

Total genomic DNA was extracted from fresh leaves using the cetyl trimethylammonium bromide method [40] at the heading stage once visibly distinct phenotypes were apparent. The total PCR reaction volume was 12.5 μL and contained the following components: 1.25 μL of 10× PCR buffer, 1 μL of 10 μmol L^− 1^ primers, 1 μL of 50 ng μL^− 1^ DNA, 0.75 μL of 25 mmol L^− 1^ MgCl_2_, 0.5 μL of 2.5 mmol L^− 1^ dNTPs, 7.9 μL ddH_2_O, and 0.1 μL of 5 U μL^− 1^ Taq DNA polymerase. The PCR reaction system was as follows: 5 min at 94 °C, followed by 35 cycles of 30 s at 94 °C, 30 s at 55 °C and 1 min at 72 °C. The PCR products were separated in a 10% polyacrylamide gel and visualized by rapid silver staining.

### Development of new Indel and SSR markers

To localize *SNFL1* to a narrower chromosomal interval, new Indel and SSR markers were designed because of the lack of known markers. The *japonica* rice Nipponbare genome sequence [1] was used as a query for a BLAST search against the entire genome sequence of *indica* rice Jinhui10. Differences in more than two nucleotides were chosen to design Indel markers, and new SSR markers were identified on the basis of differences in the length of repeat sequences of Nipponbare and Jinhui10 using SSR-Hunter 1.3. The newly designed markers are listed in Additional file 4: Table S1.

### Map-based cloning of *SNFL1*

To determine the chromosomal location of *SNFL1*, 980 F_2_ plants that showed the mutant phenotype were selected and used as a mapping population. Initial mapping was carried out using SSR markers based on 12 rice linkage maps (http://rgp.dna.affrc.go.jp). Fine mapping was performed using the newly designed Indels and SSR markers after the chromosomal region was determined by linkage analysis. The sequences of primers used in the mapping are listed in Additional file 4: Table S1.

### Vector construction

To construct the *SNFL1* complementation plasmid, a 3881-bp genomic DNA fragment containing the entire 960 bp *SNFL1* coding sequence, a 2362-bp upstream region, and a 559-bp downstream region were amplified using the primers *SNFL1*com-F and *SNFL1*com-R from WT genomic DNA. The amplified fragment was digested with *Bam*HI and *Hin*dIII, and cloned into the binary vector pCAMBIA1301. The recombinant plasmids were introduced into the *snfl1* mutant by *Agrobacterium tumefaciens*-mediated transformation as described previously [41]. The primer sequences are listed in Additional file 5: Table S2.

### GUS expression assay

To analyse the expression pattern of *SNFL1*, a ~ 3031-bp promoter fragment was cloned into the pCAMBIA1301 vector using the *SNFL1*-specific primers *SNFL1*-PF and *SNFL1*-PR to create the *PRO*_*SNFL1*_*:GUS* reporter gene construct, which was then transformed into Jinhui10 by *A. tumefaciens*-mediated transformation. GUS staining was performed on *PRO*_*SNFL1*_*:GUS* T_1_ generation transgenic plants in accordance with the method of Jefferson et al. [42]. Digital images were captured using a Canon EOS 5D Mark III camera. Primers used to clone the promoter fragment are listed in Additional file 5: Table S2.

### Subcellular localization

The coding sequence of *SNFL1* lacking the stop codon was fused to the N-terminus of the *GFP* gene under the control of the enhanced CaMV 35S promoter in the *Spe*I and *Bam*HI sites of the vector pAN580 to generate the *pSNFL1-GFP* construct. The SNFL1-GFP fusion protein was transiently expressed in rice leaf protoplasts by a previously described method [14]. Fluorescence was observed using a LSM800 confocal laser microscope (Zeiss, Jena, Germany). The primer sequences used are listed in Additional file 5: Table S2.

### Protein sequence and phylogenetic analysis

For phylogenetic analysis of SNFL1 homologs in plants, protein sequences were obtained by searching GenBank (http://www.ncbi.nlm.nih.gov/genbank/) using the SNFL1 sequence as a query. A multiple-sequence alignment was generated using ClustalX2.1. A phylogenetic tree was constructed with MEGA 5.05 using the maximum likelihood method. Topological robustness was assessed by means of a bootstrap analysis with 1000 replicates.

## Additional files

Additional file 1: **Figure S1.** The phenotype of *snfl1* mutant. (A-B) Plants of WT and *snfl1* in the field. (C) A culm of WT and *snfl1* mutant at maturing stage, vegetative leaves being removed. Arrow indicate overgrown culms. (Scale bar, 10 cm.) (D) Internodes and panicles of WT and *snfl1* mutant. (Bars = 5 cm.) (E) Statistical data of internodes in WT and *snfl1*. (F-G) Statistical data of length and width of second and third leaves between WT and *snfl1*. Means ± SD are given in E (*n* = 10), F (*n* = 50), and G (*n* = 50). **P < 0.01, ***P < 0.001 (*t*-test). (PPT 1000 kb)

Additional file 2: **Figure S2.** Genetic sequences of *LOC_Os05g50270*. The ATG and TAG codon were shaded in green, the mutant site was shaded in grey. The red box indicates the restriction enzyme cutting site at the mutant site. The primer sequences to sequence cDNA were shaded in yellow. (PPT 153 kb)

Additional file 3: **Figure S3.** Sequence verification of *SNFL1* in complement transgenic plants. Black arrows represent the peak of mutation and rescue. (PPT 96 kb)

Additional file 4: **Table S1.** Primer sequences for mapping *SNFL1*. (XLS 28 kb)

Additional file 5: **Table S2.** Primers used to construct vectors. (XLS 26 kb)

## Abbreviations

AS: Alternative splicing
BLAST: Basic Local Alignment Search Tool
COM: Complemented transgenic plant
EMS: Ethyl methanesulfonate
GFP: Green fluorescent protein
GUS: β-glucuronidase
IRGSP: International Rice Genome Sequencing Project
ORFs: Open reading frames
RT-PCR: Reverse transcription-polymerase chain reaction
SSR: Simple sequence repeat
WT: Wild type
