# Supplementary figures and images for: Short and narrow flag leaf1, a GATA zinc finger domain-containing protein, regulates flag leaf size in rice (Oryza sativa)
Source: BMC Plant Biol. 2018 Nov 9;18:273. doi: 10.1186/s12870-018-1452-9 (PMC6230254; doi:10.1186/s12870-018-1452-9)

## Slide 1
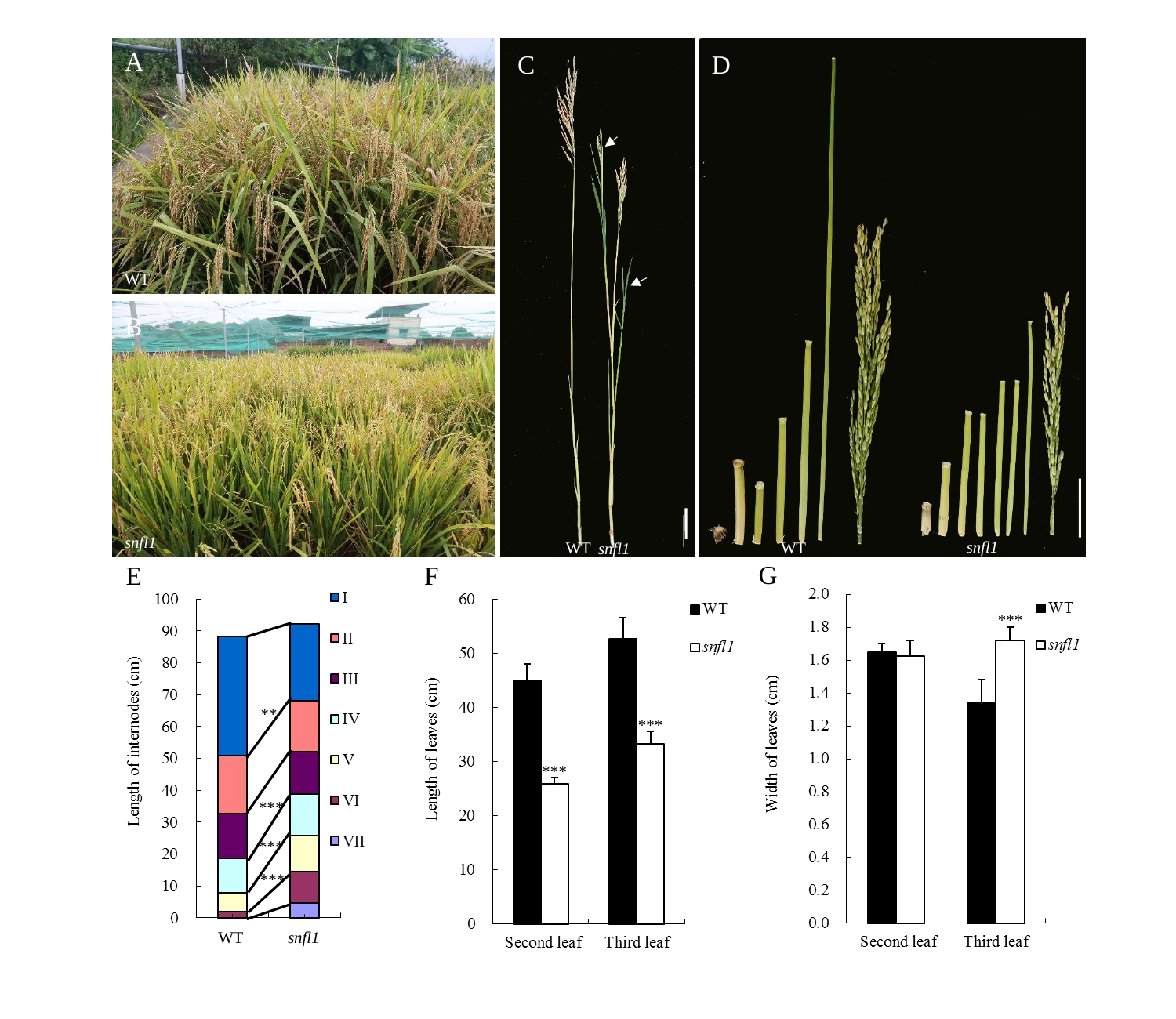

A
C
D
WT
B
snfl1
snfl1
WT
WT
snfl1
G
E
F
***
**
***
***
***
***
***
WT
snfl1

Supplement: Supplementary file 1 — Figure S1. The phenotype of snfl1 mutant. (A-B) Plants of WT and snfl1 in the field. (C) A culm of WT and snfl1 mutant at maturing stage, vegetative leaves being removed. Arrow indicate overgrown culms. (Scale bar, 10 cm.) (D) Internodes and panicles of WT and snfl1 mutant. (Bars = 5 cm.) (E) Statistical data of internodes in WT and snfl1. (F-G) Statistical data of length and width of second and third leaves between WT and snfl1. Means ± SD are given in E (n = 10), F (n = 50), and G (n = 50). **P < 0.01, ***P < 0.001 (t-test). (PPT 1000 kb) [file 12870_2018_1452_MOESM1_ESM.ppt]

## Slide 1
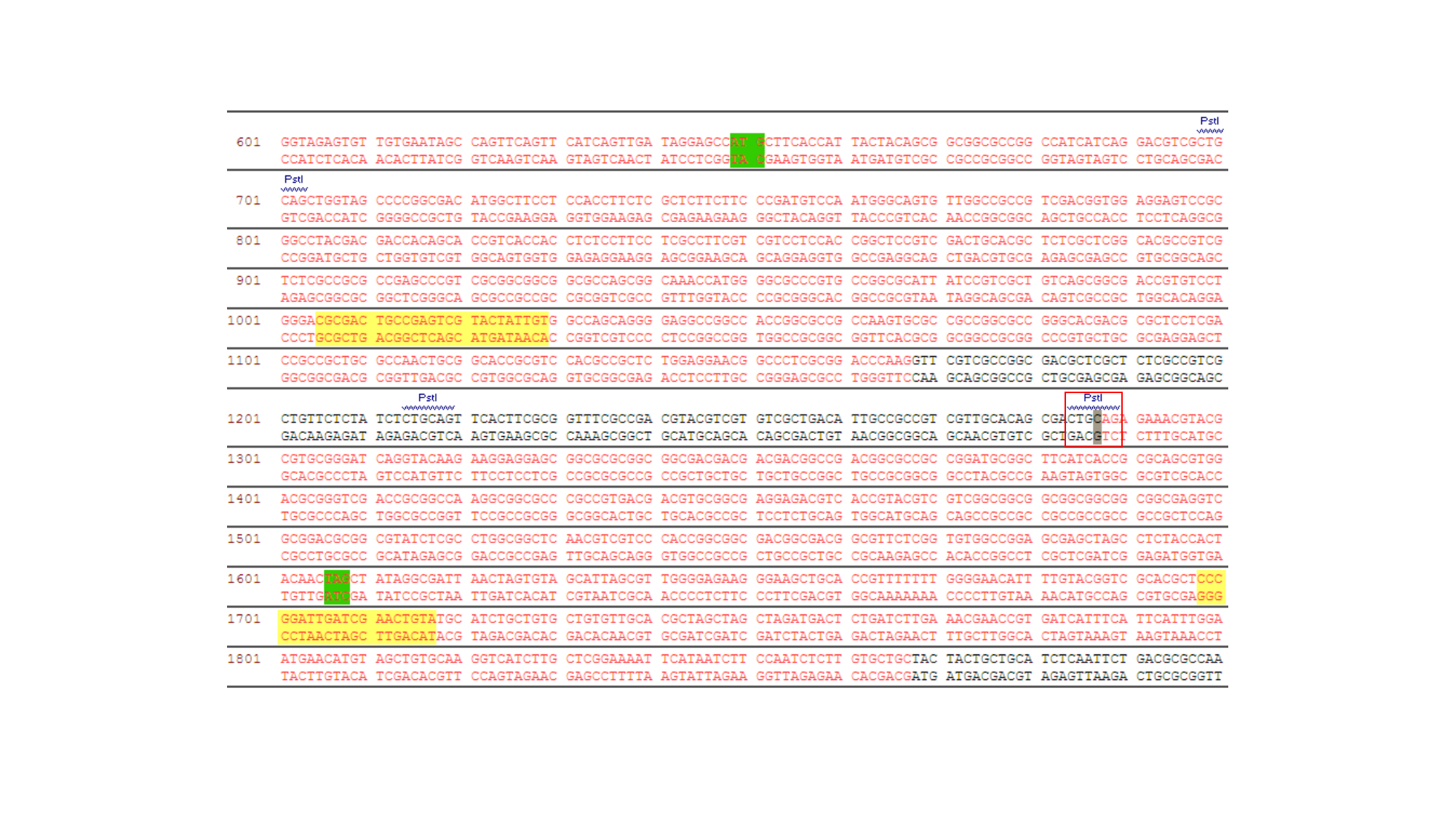

Supplement: Supplementary file 2 — Figure S2. Genetic sequences of LOC_Os05g50270. The ATG and TAG codon were shaded in green, the mutant site was shaded in grey. The red box indicates the restriction enzyme cutting site at the mutant site. The primer sequences to sequence cDNA were shaded in yellow. (PPT 153 kb) [file 12870_2018_1452_MOESM2_ESM.ppt]

## Slide 1
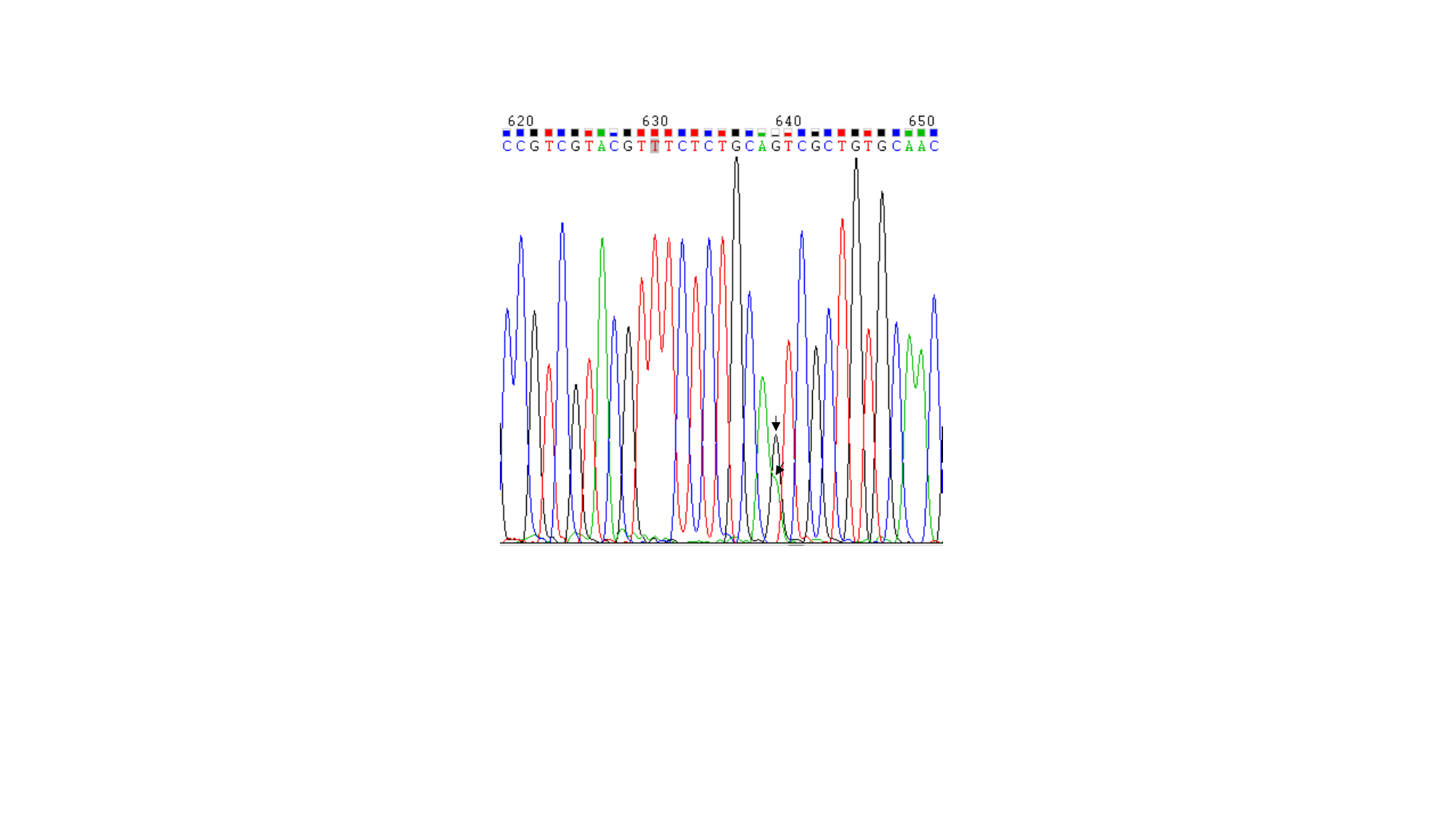

Supplement: Supplementary file 3 — Figure S3. Sequence verification of SNFL1 in complement transgenic plants. Black arrows represent the peak of mutation and rescue. (PPT 96 kb) [file 12870_2018_1452_MOESM3_ESM.ppt]
